# The Anthraquinone Derivative C2 Enhances Oxaliplatin-Induced Cell Death and Triggers Autophagy via the PI3K/AKT/mTOR Pathway

**DOI:** 10.3390/ijms25126468

**Published:** 2024-06-12

**Authors:** Yuying Li, Wei Yan, Yu Qin, Liwei Zhang, Sheng Xiao

**Affiliations:** 1 Key Laboratory of Chemical Biology and Molecular Engineering of Education Ministry, Shanxi Key Laboratory of Biotechnology, Institute of Biotechnology, Shanxi University, Taiyuan 030006, China; yanwei978615472@163.com (W.Y.); ty-qiny@kingmed.com.cn (Y.Q.); 2 Key Laboratory of Chemical Biology and Molecular Engineering of Education Ministry, Institute of Molecular Science, Shanxi University, Taiyuan 030006, China; lwzhang@sxu.edu.cn; 3 Department of Pathology, Brigham and Women’s Hospital, Harvard Medical School, Boston, MA 02115, USA; sxiao@rics.bwh.harvard.edu

**Keywords:** anthraquinone derivative C2, autophagy, multidrug resistance

## Abstract

Chemotherapy resistance in cancer is an essential factor leading to high mortality rates. Tumor multidrug resistance arises as a result of the autophagy process. Our previous study found that compound 1-nitro-2 acyl anthraquinone-leucine (C2) exhibited excellent anti-colorectal cancer (CRC) activity involving autophagy and apoptosis-related proteins, whereas its underlying mechanism remains unclear. A notable aspect of this study is how C2 overcomes the multidrug susceptibility of HCT116/L-OHP, a colon cancer cell line that is resistant to both in vitro and in vivo oxaliplatin (trans-/-diaminocyclohexane oxalatoplatinum; L-OHP). In a xenograft tumor mouse model, we discovered that the mixture of C2 and L-OHP reversed the resistance of HCT116/L-OHP cells to L-OHP and inhibited tumor growth; furthermore, C2 down-regulated the gene expression levels of *P-gp* and *BCRP* and decreased *P-gp’s* drug efflux activity. It is important to note that while C2 re-sensitized the HCT116/L-OHP cells to L-OHP for apoptosis, it also triggered a protective autophagic pathway. The expression levels of cleaved caspase-3 and Beclin 1 steadily rose. Expression of PI3K, phosphorylated AKT, and mTOR were decreased, while p53 increased. We demonstrated that the anthraquinone derivative C2 acts as an L-OHP sensitizer and reverses resistance to L-OHP in HCT116/L-OHP cells. It suggests that C2 can induce autophagy in HCT116/L-OHP cells by mediating p53 and the PI3K/AKT/mTOR signaling pathway.

## 1. Introduction

In 2018, colorectal cancer ranked third in the number of new cancer cases worldwide, accounting for 10.2%, and the number of deaths due to colorectal cancer ranked second, accounting for 880,000 [1]. The high rate of new colorectal cancer cases and the rising death rate not only bring great pain to millions of families but also massive financial loss, as well as imposing a heavy medical burden. Thus, the treatment of colorectal cancer represents one of the significant challenges for the global medical system.

The main clinical drugs currently used to treat colorectal cancer are 5-Fluorouracil (5-Fu), Oxaliplatin (L-OHP), and bevacizumab [2,3]. However, as the number of drugs used in patients increases, the ability of the drugs to inhibit tumor cells is diminished and drug resistance can develop, rendering chemotherapy ineffective. In chemotherapy for colorectal cancer, tumor cells not only acquire tolerance to a particular drug but can also develop cross-resistance to other unexposed drugs with different structures and mechanisms of action [4,5].

P-glycoprotein (P-gp; ABCB1) and breast cancer resistance protein (BCRP; ABCG2) are ATP-binding cassette transporters. These proteins can bind drug molecules within cells and utilize intracellular energy from ATP to expel the drugs from the cell, thereby decreasing the concentration of drugs in the cell. This counteracts the expected therapeutic effect and can lead to the development of drug resistance in the tumor [6]. Evodiamine has been reported to inhibit the upregulation of ABCG2 protein in drug-resistant HCT116/L-OHP colon cancer cells and to increase sensitivity to L-OHP [7]. Other studies have shown that Zuo Jin Wan, a traditional Chinese medicine, can reverse P-gp-mediated multidrug resistance in HCT116/L-OHP cells [8]. Furthermore, an active compound extracted from Australian plants has been found to inhibit the activity of BCRP, reversing tumor resistance [9]. Therefore, both BCRP and P-gp proteins represent essential targets in the reversal of drug resistance.

The PI3K/AKT/mTOR pathway plays an important role in various tumor cells through the regulation of processes such as proliferation, differentiation, survival, apoptosis, autophagy, and angiogenesis, promoting tumor progression [10,11,12,13,14]. Mammalian target of rapamycin (mTOR) is a key component of autophagy pathways. mTOR forms two complexes, mTORC1 and mTORC2, of which mTORC1 inhibits autophagy while the function of mTORC2 is unclear [15]. However, there is evidence for the involvement of mTORC2 in cell survival and metabolism, and it has been shown that mTORC1 can negatively regulate mTORC2 through S6K [16]. The activation of mTOR inhibits autophagy [17]. Curcumin has been found to down-regulate the expression of PI3K, p-AKT, and p-mTOR to induce autophagy in renal cells, thereby improving renal function [18]. A study found that inhibition of the PI3K/AKT/mTOR pathway mediated both apoptosis and autophagy in AGS gastric cancer cells [19], while Yang et al. found that inhibition of the PI3K, AKT, and mTOR signaling pathways induced apoptosis and autophagy in HepG2 liver cancer cells [20].

**Figure 1:**
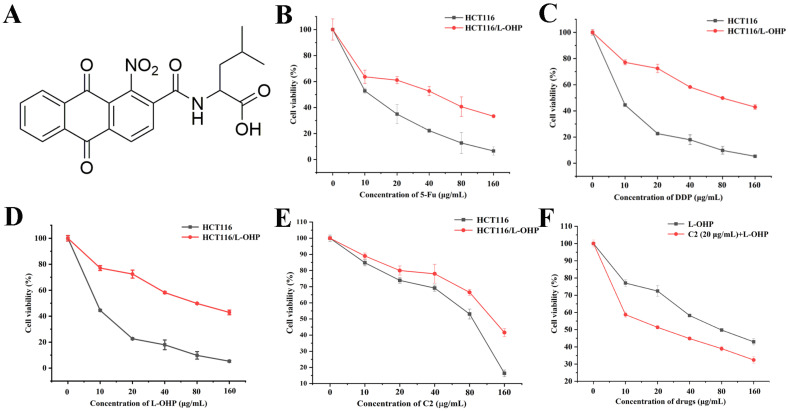
Multidrug resistance to HCT116/L-OHP cells. (**A**) Chemical structure of C2. (**B**–**D**) Different concentrations of L-OHP, DDP, and 5-Fu (0, 10, 20, 40, 80, 160 μg/mL) were applied to sensitive cells HCT116 and drug-resistant cells HCT116/L-OHP for 48 h, and the absorbance value was detected by MTT. (**E**) Different concentrations of C2 treated on HCT116 and HCT116/L-OHP cells for 48 h, cell viability was measured by MTT assays. (**F**) C2 (20 μg/mL combined with L-OHP and L-OHP alone) treatment on drug-resistant cells for 48 h, MTT detected cell viability. All the experiments were repeated five times. Data represent the mean ± standard.

Anthraquinone is a polycyclic organic compound composed essentially of three benzene rings with two ketone groups in the middle ring. The most common natural anthraquinone is 9, 10-Anthraquinone, which is present not only in many higher plants but also in smaller amounts in bacteria and fungi. Anthraquinone was used as a natural dye in ancient China and is found in Chinese herbs such as *Rheum palmatum*, *Polygonum multiflorum*, and *Polygonum cuspidatum* [21,22]. Anthraquinones have a broad spectrum of biological activities, including anti-bacterial [23], anti-viral [24], anti-diabetic [25], and anti-tumor properties [26], among others, of which anti-cancer activity has attracted the most attention. An anthraquinone compound extracted from *Morinda citrifolia* L. can inhibit the growth of MDA-MB 231 and MCF-7 breast cancer cells, as well as induce cellular apoptosis to inhibit the development of tumors [27]. Dai et al. reported that emodin could down-regulate VEGFR2 expression in HCT116 colorectal cancer cells, thereby inhibiting cell invasion and xenograft growth in vivo [28]. In addition, the combination of anthraquinone derivatives with chemotherapy drugs for cancer treatment offers great potential. Reports have shown that aloe-emodin inhibits energy metabolism in MCF-7/ADR breast cancer cells, leading to reduced intracellular ATP levels and thereby affecting the outward flow of the P-gp protein; the combination of aloe-emodin with adriamycin was found to increase the response of drug-resistant cells to doxorubicin and reverse drug resistance in MCF-7/ADR cells [29]. Guo et al. demonstrated that the association of emodin and gemcitabine could down-regulate both protein and mRNA expression of MDR/P-gp, MRP1, and MRP5 in drug-resistant PANC-1 pancreatic cell xenograft tumors, thereby promoting the anti-tumor action of gemcitabine [30]. Anthraquinone compounds belong to a class of classic organic tricyclic compounds with highly promising anti-tumor effects. We previously synthesized a series of anthraquinone derivatives and found that one anthraquinone derivative, termed C2 (Figure 1A), had the most potent inhibitory effect on HCT116 colon cancer cells, promoting both apoptosis and autophagy in these cells [31].

L-OHP is a chemotherapy drug used to treat colorectal cancer; however, its use has been limited by severe neurotoxicity and drug resistance [32]. To address this issue, researchers have combined natural compounds with L-OHP to reduce the L-OHP dosage and increase the responsiveness of resistant cells to L-OHP. Nobiletin has been reported to enhance the anti-tumor effect of L-OHP on colon cancer cells and to induce apoptosis [33], while Poria cocos combined with L-OHP significantly suppresses the migration and invasion of stomach cancer cells, as well as altering their morphology [34]. Compound sophora was found to overcome drug tolerance in L-OHP and 5-Fu-resistant colon cancer cells by down-regulating P-gp and ABCG2 and up-regulating ANXA1 [35].

The anthraquinone derivative C2 has been found to inhibit the multiplication of colon cancer cells and induce cell apoptosis [31]. Whether the association of C2 and L-OHP can target colon cancer cells that are resistant to L-OHP and enhance the therapeutic effect of L-OHP remains unclear. Therefore, we aimed to investigate the suppressive activity of C2 when applied in combination with L-OHP on HCT116/L-OHP cells both in vivo and in vitro, together with its potential underlying mechanisms.

## 2. Results

### 2.1. Multidrug Resistance in HCT116/L-OHP Cells

Colon cancer is highly susceptible to the development of drug resistance during chemotherapy. HCT116 and HCT116/L-OHP cells were treated with DDP, L-OHP, and 5-Fu for 48 h. The three drugs showed dose-dependent inhibitory effects in both HCT116/L-OHP and HCT116 cells (Figure 1B–D). The IC_50_ values of L-OHP in HCT116/L-OHP and HCT116 cells were found to be 8.47 and 86.32 μg/mL, respectively (Table 1). The resistance index (RI) of HCT116/L-OHP cells to L-OHP was 10.19, while the RI value to DDP was 8.07 and to 5-Fu was 3.56 (Table 1). It can be concluded that HCT116/L-OHP cells have multidrug resistance, and are highly resistant not only to L-OHP but also to DDP and 5-Fu.

### 2.2. C2 Enhanced the Inhibitory Effect of L-OHP on Proliferation Both In Vitro and In Vivo

**Figure 2:**
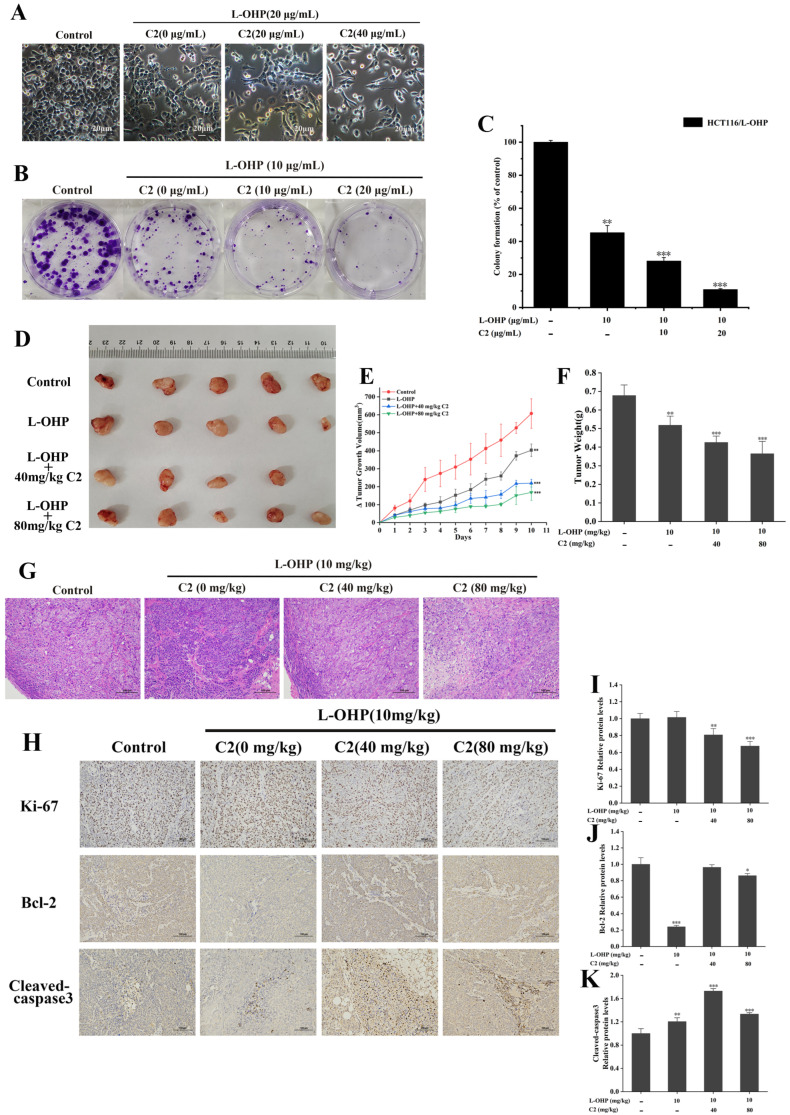
Effects of C2 combined with L-OHP on colon cancer in vivo and in vitro. (**A**) C2 reverses the effect of HCT116/L-OHP cells on morphology after C2+L-OHP treatment for 48 h, cell morphology was observed with an inverted microscope. (**B**,**C**) Colony formation of HCT116/L-OHP cells after C2+L-OHP treatment for 7 days. (**D**–**F**) C2+L-OHP inhibits tumor growth in vivo. (**E**) Δ tumor growth volume from 4 groups. (**F**) Tumor weight from 4 groups. (**G**) H&E staining analysis of pathological features of tumors from 4 groups. Bar = 100 μm. (**H**–**K**) IHC analysis of Ki67, Bcl-2, and cleaved caspase 3 expression levels of tumors from 4 groups. Bar = 100 μm. All data represent the mean ± standard deviation of three independent experiments. * *p* < 0.05, ** *p* < 0.01, *** *p* < 0.001.

To investigate whether C2 could reverse the resistance of HCT116/L-OHP cells to L-OHP, the cells were treated with different concentrations of C2 for 48 h, observing that as the C2 concentrations increased, the viability of the cells decreased (Figure 1E). The doses of 20 and 40 μg/mL, at which C2 inhibition of cell viability was less than 20%, were selected as the reversal dose. HCT116/L-OHP cells were incubated with 20 μg/mL of C2 and different concentrations (0, 10, 20, 40, 80, and 160 μg/mL) of L-OHP for 48 h. The IC_50_ value of C2+L-OHP in HCT116/L-OHP cells was 24.28 μg/mL, while the IC_50_ of L-OHP in HCT116/L-OHP cells was 86.32 μg/mL (Figure 1F). It is thus apparent that C2 increased the sensitivity of HCT116/L-OHP cells to L-OHP. HCT116/L-OHP cells were then incubated with C2 and L-OHP for 48 h, observing no significant changes in the cell morphology of the L-OHP group compared with the control group. Combined treatment of cells with C2 and L-OHP, together with increased C2 concentration, resulted in reduced cell densities and collapse of the cells (Figure 2A). Similarly, increased C2 concentrations led to significant reductions in the clone formation rate of HCT116/L-OHP cells (Figure 2B,C). In the tumor-bearing mouse model, C2 combined with L-OHP successfully inhibited tumor growth (Figure 2D–F). Immunohistochemical analysis showed that C2 had a dose-dependent effect on the expression of Ki67; however, the expression of Bcl-2 and cleaved Caspase 3 was much higher in the L-OHP+C2 group than in the L-OHP group (Figure 2H–K). Histological analysis using HE staining showed that compared with the control and L-OHP groups, cell boundaries in the combined treatment group were blurred, accompanied by karyopyknosis and fragmentation of the nuclei (Figure 2G).

### 2.3. C2 Affects Drug Efflux from HCT116/L-OHP Cells and Drug-Resistant Proteins

**Figure 3:**
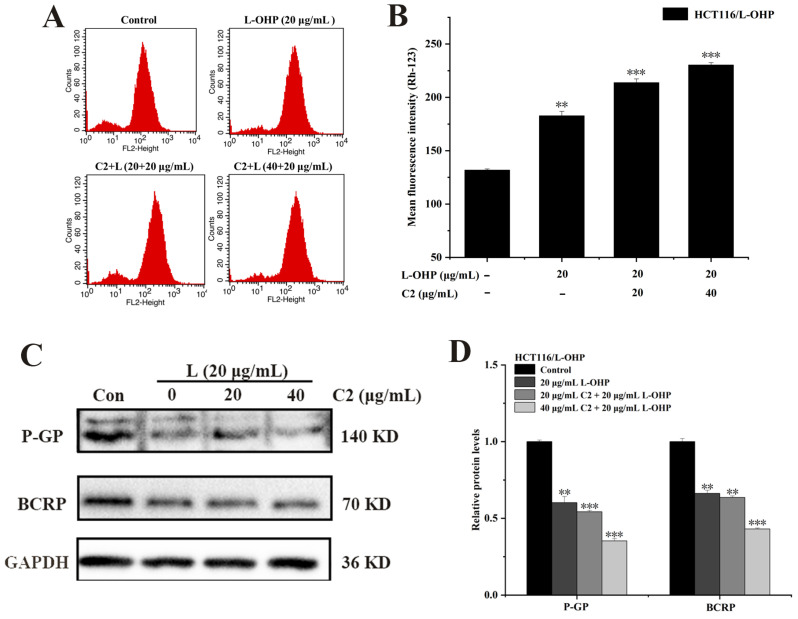
Effects of C2 combined with L-OHP on P-gp and BCRP. (**A**,**B**) Rhodamine-123 staining was analyzed for the efflux activity of P-gp after C2+L-OHP treatment by flow cytometry. (**C**,**D**) Expression of P-gp and BCRP was examined by Western blotting analysis. All data represent the mean ± standard deviation of three independent experiments. ** *p* < 0.01, *** *p* < 0.001.

Intracellular P-glycoprotein (P-gp) is a multi-specific drug efflux transporter. It plays a crucial role in drug resistance and management [36]. P-gp can bind to rhodamine-123, allowing the detection of the distribution of drug-resistant cells by flow cytometry [37]. P-gp can remove the drugs from the cells after binding them, thus preventing the drugs from taking effect and reducing the overall therapeutic effect [38]. In this study, it was found that C2 (0, 20, and 40 μg/mL) when combined with L-OHP, was effective in treating HCT116/L-OHP cells; it was found that as the C2 concentration increased, the intensity of the rhodamine-123 fluorescence increased (Figure 3A,B), indicating reduced efflux of the P-gp protein. It can thus be concluded that C2 can reduce drug efflux from drug-resistant HCT116/L-OHP cells.

To investigate the effect of C2 on the expression of proteins associated with drug resistance in HCT116/L-OHP cells, HCT116/L-OHP cells were treated with different concentrations of C2 (0, 20, and 40 μg/mL) combined with L-OHP. Western blotting was used to determine the expression of P-gp and BCRP in protein samples. It was found that the expression of P-gp and BCRP decreased gradually as the C2 concentration increased (Figure 3C,D). Therefore, C2 can reduce the expression of proteins associated with drug resistance in HCT116/L-OHP cells. Taken together, C2 can affect the promotion of drug efflux by P-gp and inhibit the expression of the drug-resistance-related proteins P-gp and BCRP.

### 2.4. C2 Enhances L-OHP-Induced Apoptosis and Autophagy

**Figure 4:**
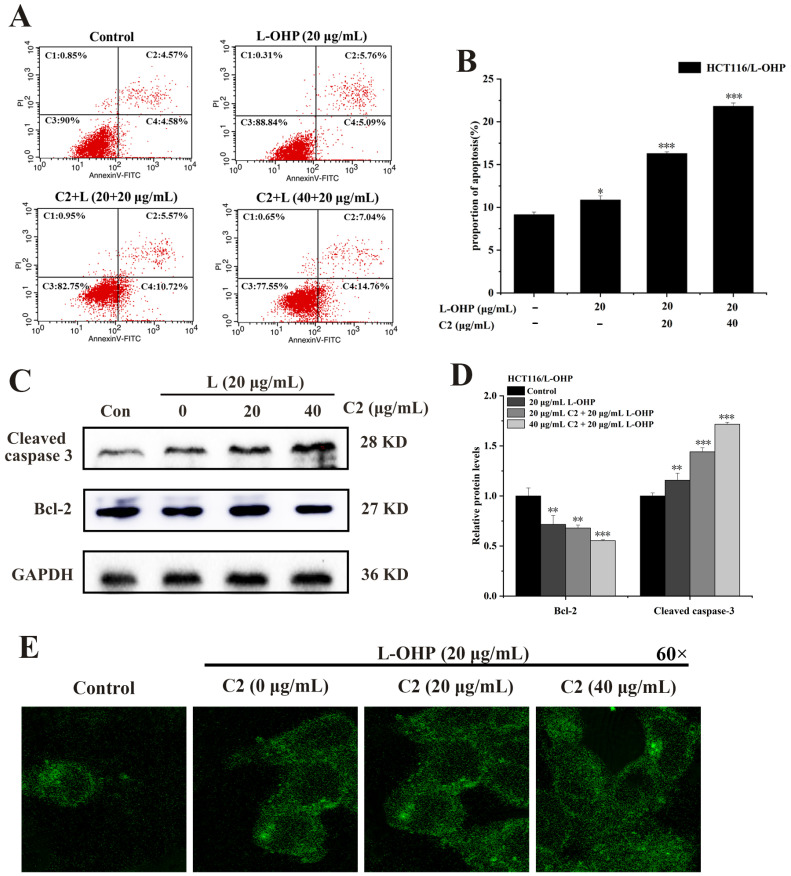
C2 enhanced L-OHP-induced apoptosis and autophagy. (**A**,**B**) Flow cytometry was used to analyze the effect of C2 on L-OHP-induced apoptosis. (**C**,**D**) Expression of Bcl-2 and cleaved caspase 3 was examined by Western blotting analysis. (**E**) MDC staining analyzed the C2-enhanced L-OHP-induced autophagy by the laser confocal microscope (60×). All data represent the mean ± standard deviation of three independent experiments. * *p* < 0.05, ** *p* < 0.01, *** *p* < 0.001.

To investigate whether C2 increased the sensitivity of HCT116/L-OHP cells to L-OHP and further enhanced L-OHP-induced apoptosis, cells were treated with C2 (0, 20, and 40 μg/mL) and L-OHP for 48 h and stained with Annexin-V and PI, after which the number of apoptotic cells was assessed using flow cytometry. It was found that the number of apoptotic cells was positively correlated with C2 concentration compared with the control group. The rate of apoptosis in the C2 (20, 40 μg/mL) plus L-OHP group was higher than that in the group treated with L-OHP alone (Figure 4A,B). The expression of Bcl-2 and cleaved Caspase 3 was then assessed in the cells, finding that compared with the control group, the levels of cleaved Caspase 3 increased gradually in correspondence with the C2 concentration, while those of Bcl-2 decreased (Figure 4C,D). This suggests that C2 can increase the sensitivity of HCT116/L-OHP cells to L-OHP by enhancing L-OHP-induced apoptosis.

MDC (monodansylcadaverine) is an autofluorescent compound that can specifically label autophagic vesicles [39]; thus, MDC was used to determine autophagic flux, with cells incubated in a medium supplemented with different concentrations of C2 (0, 20, and 40 μg/mL) and L-OHP for 48 h to observe changes in autophagic vesicles in the cells. It was observed that the area of green fluorescence gradually increased as the C2 concentration increased (Figure 4E). These results indicated that the anthraquinone derivative C2 enhanced L-OHP-induced autophagy.

### 2.5. C2 Enhances L-OHP-Induced Autophagy by Modulating the PI3K/AKT/mTOR Pathway

**Figure 5:**
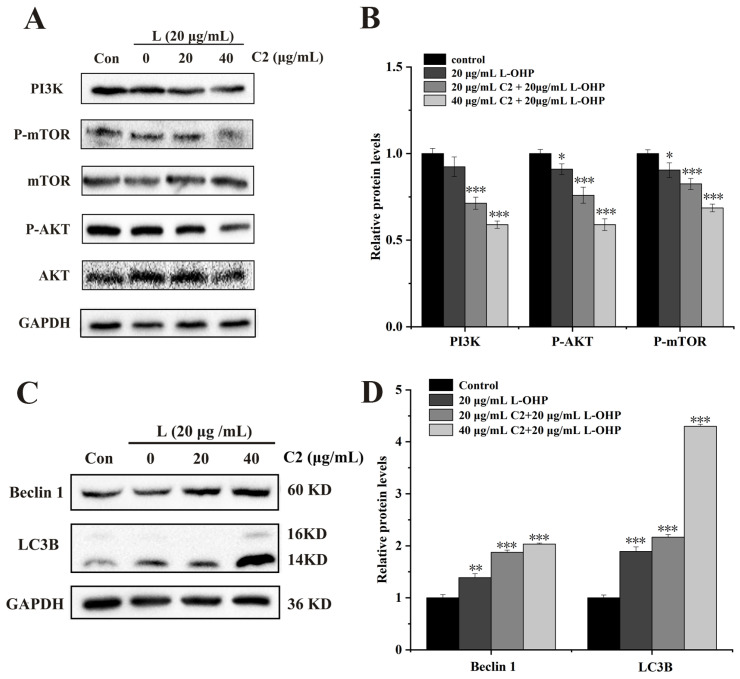
C2 enhances L-OHP-induced autophagy by modulating PI3K/AKT/mTOR pathway. (**A**,**B**) Expression of PI3K, AKT, mTOR, p-AKT, and p-mTOR were examined by Western blotting analysis. (**C**,**D**) Expression of Beclin 1 and LC3B was examined by Western blotting analysis. All data represent the mean ± standard deviation of three independent experiments. * *p* < 0.05, ** *p* < 0.01, *** *p* < 0.001.

mTOR is the core hub of autophagy and the PI3K/AKT/mTOR pathway is closely involved in cell proliferation, apoptosis, and autophagy [40,41]. Therefore, C2 (0, 20, and 40 μg/mL) and L-OHP were combined to evaluate the expression of PI3K/AKT/mTOR pathway proteins. As shown in Figure 5, the expression of PI3K decreased with increased C2 concentration. In terms of the expression of proteins downstream of PI3K, no change was observed in the levels of mTOR and AKT, while those of p-mTOR and p-AKT decreased gradually (Figure 5A–D). Then, the expression of Beclin 1 and the LC3II/I ratio increased in correspondence with the C2 concentration. Therefore, these data suggest that C2 enhances L-OHP-induced autophagy through the PI3K/AKT/mTOR signaling pathway.

### 2.6. The Combination of C2 and L-OHP Induces Protective Autophagy

**Figure 6:**
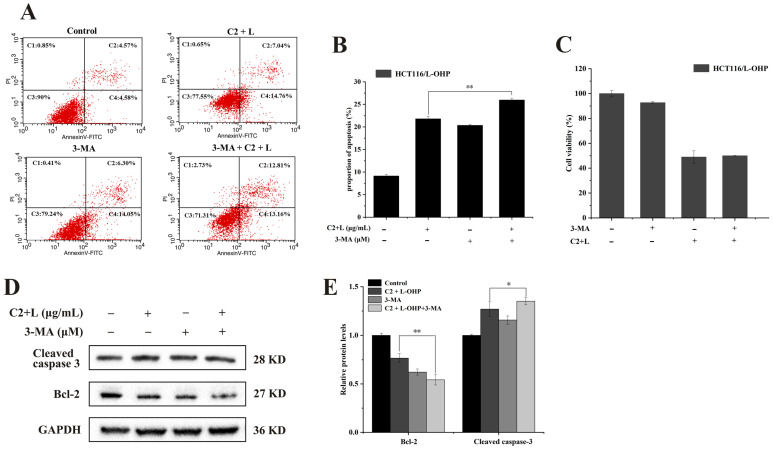
The combination of C2 and L-OHP induced protective autophagy. (**A**,**B**) Flow cytometry was used to analyze the effect of 3-MA (100 μΜ) on C2+L-OHP-induced apoptosis. (**C**) Cell viability of 3-MA combined with C2+L-OHP treatment by MTT. (**D**,**E**) Expression of Bcl-2 and cleaved caspase3 was examined by Western blotting analysis after 3-MA combined with C2+L-OHP treatment. All data represent the mean ± standard deviation of three independent experiments. * *p* < 0.05, ** *p* < 0.01.

3-Methyladenine (3-MA) is an autophagy inhibitor, inhibiting both class III PI3K and the formation of autophagic vesicles [42]. To explore the effects of the C2-L-OHP combination in inducing apoptosis or autophagy, 3-MA and C2+L-OHP were co-incubated with cells. As shown in Figure 6, after the treatment of cells with 3-MA and/or C2+L-OHP, cell viability was lower in cells treated with 3-MA together with C2+L-OHP than in either the C2+L-OHP or 3-MA groups (Figure 6C). Flow cytometry was then used to examine apoptosis. It was found that the rate of apoptosis in cells treated with 3-MA together with C2+L-OHP was higher than that of those treated with C2+L-OHP or 3-MA alone (Figure 6A,B). Furthermore, expression of Bcl-2 was decreased, while that of cleaved caspase 3 was increased (Figure 6D,E). This evidence indicated that the cells switched to apoptosis when autophagy was inhibited, demonstrating that C2 enhancement of L-OHP-induced autophagy was protective and promoted cell survival.

## 3. Discussion

L-OHP is used as a drug to treat colon cancer but its efficacy is reduced due to multidrug resistance in tumor cells. It is thus urgent to find an agent that can counteract this resistance. Here, to address this problem, a combination of L-OHP and C2 was used to assess its potential for reducing drug resistance in HCT116/L-OHP drug-resistant colorectal carcinoma cells. This study first verified that HCT116/L-OHP cells were resistant not only to L-OHP but also to DDP and 5-Fu, indicating the presence of multidrug resistance. It was then observed that the viability of HCT116/L-OHP cells decreased as the C2 concentration increased. According to the IC_50_ value of the C2-L-OHP combination, C2 concentrations of 20 and 40 μg/mL were selected as effective doses for subsequent experiments. The combination of C2 and L-OHP reduced cell viability in correspondence with increasing C2 concentration. Microscopic observations showed increased spaces between cells, with reduced cell densities and volumes. Likewise, colony formation experiments indicated significantly reduced colony numbers as the C2 concentration in the combined treatment increased. Therefore, C2 can inhibit the proliferation of HCT116/L-OHP cells.

P-gp and BCRP are ATP-binding cassette transporters expressed in the cell membrane that can use ATP to transport drugs or natural toxins from the cell, thus playing a role in cell protection [43,44]. This represents one of the reasons for drug resistance in tumor cells. The efflux activity of P-gp was assessed by flow cytometry. The combined C2 and L-OHP treatment resulted in increased rhodamine-123 fluorescence within the cells, indicating reduced P-gp efflux activity. Similarly, the expression levels of P-gp and BCRP were also reduced in a dose-dependent manner. Thus, it is inferred that C2 reversed L-OHP resistance in HCT116/L-OHP cells.

Previous studies have shown that C2 can induce the apoptosis of HCT116 cells [31]. In terms of whether C2 can enhance the sensitivity of HCT116/L-OHP cells to L-OHP, the findings of the present study suggest that the apoptotic cells increased on co-treatment with C2 and L-OHP compared with L-OHP alone. Moreover, the expression levels of cleaved caspase 3 increased and Bcl-2 decreased after treatment with the combined regimen. These results indicate that C2 can enhance L-OHP-induced apoptosis, and thus suggest that C2 can be used as a sensitizer of L-OHP to reverse the resistance of HCT116/L-OHP cells to L-OHP. It was also observed that C2 enhanced L-OHP-induced autophagy and autophagic flux with increased C2 concentrations.

Although the above conclusion indicates that C2 can enhance L-OHP-induced apoptosis and autophagy, the mechanism is unclear. It has been reported that the PI3K/AKT/mTOR signaling pathway is closely associated with the proliferation, apoptosis, and autophagy of various tumor cells [41]. A previous study found that activation of the PI3K/AKT/mTOR pathway by the elevated expression of PDK1 in liver cancer cells enhanced resistance to radiotherapy in these cells, as well as promoting both cell invasion and survival [45]. Sun et al. found that aloin combined with metformin inhibited the proliferation and invasion of hepatocellular carcinoma cells, and induced apoptosis and autophagy through the PI3K/AKT/mTOR signaling pathway, enhancing the anti-cancer effect [46]. Zheng et al. demonstrated that emodin-induced apoptosis and autophagy of human hepatocytes through PI3K/AKT/mTOR decreased cell viability and increased the rate of apoptosis after inhibition of autophagy through 3-MA, indicating that autophagy induced by emodin played a protective role [47]. Nigrosporin B, an anthraquinone derivative, induces protective autophagy in cervical cancer cells by inhibiting the phosphorylation of PI3K, AKT, and mTOR [48].

The findings of the present study indicate that the combination of C2 with L-OHP can down-regulate the expression levels of PI3K, p-AKT, and p-mTOR, thus inhibiting the PI3K/AKT/mTOR pathway; meanwhile, the expression of LC3B and Beclin 1 increased, indicating the induction of autophagy. These results are consistent with those of the studies described above, and it is thus hypothesized that the combination of C2 and L-OHP induces autophagy through the PI3K/AKT/mTOR pathway. Interestingly, it was also found that when 3-MA and C2+L-OHP were used to co-treat cells, the viability of the cells decreased while the rate of apoptosis increased, together with reduced expression of Bcl-2 and increased expression of cleaved caspase 3. This suggests that cells switch to apoptosis when autophagy is inhibited, which represents protective autophagy. Together, the results suggest that C2 enhances L-OHP-induced apoptosis, and the resultant stress activates autophagy through the PI3K/AKT/mTOR pathway to promote cell survival.

The in vivo findings were then verified in tumor-bearing mice, finding that both low and high doses of C2 combined with L-OHP blocked the growth of xenograft tumors. HE staining of tumor sections showed normal cell morphology in both the control and L-OHP groups, with a tight arrangement of the cells. In contrast, in the combined treatment group, the cell boundaries were blurred, with pyknotic, broken, and differently sized nuclei. Immunohistochemical analysis of the tumor tissue showed that the levels of both Ki67 and cleaved caspase 3 were decreased in the combination group, while that of Bcl-2 did not change significantly.

In summary, autophagy itself is a protective mechanism associated with drug resistance. It was found that, even in the case of a combination of drugs, resistant cells could stimulate autophagy along with apoptosis. Cells might switch to apoptosis when autophagy is inhibited, and autophagy was shown to be activated through the PI3K/AKT/mTOR pathway, which is an interesting phenomenon; however, its in-depth study needs further exploration. We hope that the specific conditions and mechanisms of its occurrence can be clarified in future work.

## 4. Materials and Methods

### 4.1. Reagents and Chemicals

1-nitro-2-acyl anthraquinone-leucine (C2) was synthesized from 2-methyl anthraquinone (Energy Chemical, Shanghai, China) in a four-part reaction [31]. MTT, DDP, and 5-Fu (Solarbio, Beijing, China). RPMI-1640 (Gibco, Waltham, MA, USA). FBS (Tianhang Biological, Zhejiang, China). Oxaliplatin and Sodium carboxymethyl cellulose (Aladdin, Shanghai, China). Rhodamine-123 (Keygen Biotech, Jiangsu, China). DMSO (Sangon Biotech, Shanghai, China). Isoflurane (RWD Life Science, Shenzhen, China). HE dye solution (Sinopharm Chemical Reagent, Shanghai, China). 3-MA (Topscience, Torrance, CA, USA). Ki67 antibody (Servicebio, Wuhan, China). P-gp, BCRP, and p-AKT antibody (Proteintech, Wuhan, China). LC3B antibody (Cell Signaling Technology, Danvers, MA, USA). Bcl-2 antibody (Beyotime Biotechnology, Shanghai, China). Cleaved caspase-3 antibody (Bioss, Beijing, China). PI3K and AKT antibody (Wanleibio, Shenyang, China). mTOR and p-mTOR antibody (Abcam, Cambridge, UK).

### 4.2. HCT116/L-OHP Cell Culture

HCT116/L-OHP cells were purchased from Shanghai Meixuan Biological Technology. The HCT116/L-OHP cell lines were maintained by treatment at continuous exposure to the rising concentrations of L-OHP. Briefly, HCT116/L-OHP cells were cultured in RPMI-1640 (10% FBS), which were then treated with L-OHP (5, 7, and 10 μg/mL).

### 4.3. Cell Proliferation Assay

Cell viability was evaluated by MTT assay. In brief, cells were seeded to 96-well plates overnight at a density of 10^4^ cells/well. Cells were treated in different concentrations of L-OHP, DDP, and 5-Fu (10, 20, 40, 80, and 160 μg/mL) at 37 °C, 5% CO_2_ for 24 h and 48 h, respectively. Then, 20 μL of 5 mg/mL MTT was added to each hole and allowed to react for 4 h. The supernatant was removed, and the formazan crystals were dissolved by DMSO. The absorbance was measured at 490 nm by a microplate reader.

### 4.4. Cell Colony Formation Assay

Cells were collected at the logarithmic growth phase and added to 6-well plates at a density of 3 × 10^3^ cells/well. Cells were cultured at 5% CO_2_ and 37 °C for 24 h. A total of 10 μg/mL of L-OHP and C2 (0, 10, 20 μg/mL) were added to each well and co-incubated for a week. Next, the media were removed and washed with 1 × PBS twice, 1 mL pre-cooling methanol was added to each well, and after 5 min, methanol was removed and cells were stained by crystal violet dye. Then, the dye was removed and washed with distilled water 3 times. Finally, the number of colonies was counted under an inverted microscope.

### 4.5. Cell Apoptosis Assay

An apoptosis assay (Annexin V/PI double-staining method) was used alongside flow cytometry. In brief, after 48 h of culture, the 5 × 10^5^ cells were digested with trypsin without EDTA, washed once with PBS, and then added to Annexin V-FITC staining solution. To mix the cells, PI dye was added, and the cells were incubated at room temperature for 10 min in the dark. After that, cells were filtered through a 400-mesh cell screen and then detected using flow cytometry.

### 4.6. Cell Autophagy Assay

In brief, the cell suspension was diluted to 1 × 10^3^ cells/well and added to a 6-well plate with a cover slide. After the cells were attached, the medium was discarded, and 1 mL PBS was added to wash the cells twice. A total of 1 mL of paraformaldehyde was added into each well, fixed for 5 min, and 1 mL of MDC dye solution was added into each well, incubated at room temperature, and placed in the dark for 30 min. Afterward, the dye solution was removed, and the cells were cleaned with PBS twice. The cover slide was removed, and a drop of fluorescent quencher was placed on it. The cover slide was picked out of the 6-well plate with a needle and put upside down on the slide. The slide was sealed with nail oil. Observations under a laser confocal microscope and photographs were taken.

### 4.7. Measurement of Intracellular P-Glycoprotein Efflux Activity

The P-glycoprotein, a multidrug-resistant protein, can be integrated with rhodamine-123. The intensity of rhodamine-123 was measured by flow cytometry analysis. Briefly, HCT116/L-OHP cells were treated with trypsin and centrifuged, cells in 6-well plates were resuspended in complete medium and stained with rhodamine-123 (5 μg/mL) at 5% CO_2_ and 37 °C for 30 min. Then, cells were centrifuged and washed twice with serum-free medium. Finally, cells were resuspended in serum-free medium and cultured for 1 h at 5% CO_2_ and 37 °C. The intensity of rhodamine-123 was detected using flow cytometry.

### 4.8. Xenograft Tumor Models

Male SPF-grade BALB/C nude mice (5–6 weeks, 18–20 g) were purchased and bred at the Experimental Animal Center of Shanxi Provincial People’s Hospital. All animals received humane care in compliance with the “Guide for the Care and Use of Laboratory Animals” published by the National Institutes of Health (NIH Publication, 8th edition, 2011) and the Animal Research: Reporting In Vivo Experiments (ARRIVE) guidelines.

The animals were anesthetized with isoflurane, and HCT116/L-OHP cells (2 × 10^6^) were injured subcutaneously into the left forelimb of nude mice. When the xenograft tumor reached about 130 mm^3^, HCT116/L-OHP tumor-bearing mice were randomly divided into the control, L-OHP, and L-OHP+C2 (40 mg/kg, 80 mg/kg) groups (*n* = 5 per group). L-OHP (10 mg/kg) was administered to the mice every 2 days once—5 times—via intraperitoneal injection, and C2 (40 mg/kg, 80 mg/kg) was administered to the mice once a day—10 times—via gavage. At the same time, the control group received vehicles only at the same frequency. Tumor volume was measured every day. The tumor volume was computed with the following formula: tumor volume (mm^3^) = 1/2 × tumor length × tumor width^2^, Δ tumor volume (mm^3^) = tumor volume of the day − tumor volume of the day before. At the end of the experimental period, the xenograft tumor tissues were removed aseptically, and the tumor weight was recorded for further study.

### 4.9. Hematoxylin–Eosin (HE) Staining

Briefly, a small piece of tumor tissue was immersed in an appropriate amount of 4% paraformaldehyde to fix it, then it was embedded in paraffin and sliced. Afterward, the sections were deparaffinized, rehydrated, stained with hematoxylin for 5 min and with eosin for 5 min, dehydrated with ethanol solutions at ascending concentrations, transparentized with xylene, sealed with neutral resin, and observed under a microscope.

### 4.10. Immunohistochemical Staining

The sections were deparaffinized with xylene and rehydrated with ethanol. After inhibiting endogenous peroxidase using 3% H_2_O_2_ in PBS, the sections were rinsed with PBS and then incubated with rabbit polyclonal antibodies against Ki67, Bcl-2, and cleaved-caspase 3 at 4 °C overnight, and the secondary antibody for 50 min at room temperature. Reaction products were visualized following incubation with diaminobenzidine as a chromogen and counterstaining with hematoxylin.

### 4.11. Western Blotting

After the cells were treated with the indicated concentrations of C2 for 48 h, the cells were harvested and washed twice with PBS. Then, the cells were resuspended in a lysis buffer with protease inhibitors, transferred to a 1 mL tube, and lysed for 30 min in an ice bath. Cells were collected and centrifuged (12,000 rpm, 4 °C, 15 min), the supernatant was collected, and the concentration of total protein was determined using a BCA protein quantitation kit. Proteins were separated by SDS-PAGE and transferred to PVDF membranes, blocked in blocking buffer (with 5% nonfat dry milk), and incubated with primary antibody at 4 °C overnight. After washing three times with TBST, the membranes were incubated with a secondary antibody for 1 h at room temperature. After rewashing membranes with TBST three times, protein bands were visualized using an enhanced chemiluminescence (ECL) kit. Finally, in the gel imaging system, the protein bands were scanned, and the pictures were imported into the Image J software (https://imagej.net/ij/download.html) for gray analysis. The gray value ratio of the target protein–loading control was regarded as the relative expression of target proteins.

### 4.12. Statistical Analysis

All data represent the mean ± standard deviation (SD) values. Statistical significance was assessed using one-way analysis of variance (ANOVA) followed by Tukey’s HSD test for post hoc multiple comparisons using IBM SPSS statistical software (https://www.ibm.com/spss). Differences were considered statistically significant at *p* < 0.05 or *p* < 0.01 as indicated.

## 5. Conclusions

We demonstrated that C2 reversed the resistance of HCT116/L-OHP to L-OHP by inhibiting the efflux activity of P-gp and down-regulating the expression levels of P-gp and BCRP. C2 enhanced the L-OHP-induced apoptosis and blocked tumor growth when in combination with L-OHP, suggesting that C2 was a sensitive agent of L-OHP in HCT116/L-OHP cells. Furthermore, C2 induced protective autophagy through the PI3K/AKT/mTOR pathway.

## Figures and Tables

**Table 1:** Resistance index of HCT116/L-OHP cells and IC_50_ of HCT116 and HCT116/L-OHP cells with L-OHP, DDP, and 5-Fu.

	HCT116	HCT116/L-OHP	Resistance Index
L-OHP IC_50_	8.47 μg/mL	86.32 μg/mL	10.19
DDP IC_50_	17.22 μg/mL	138.94 μg/mL	8.07
5-Fu IC_50_	11.15 μg/mL	41.75 μg/mL	3.56

## Data Availability

The data and materials used to support the findings of this study are available from the corresponding author (Yuying Li) upon request.
