# Correction: Li et al. The Anthraquinone Derivative C2 Enhances Oxaliplatin-Induced Cell Death and Triggers Autophagy via the PI3K/AKT/mTOR Pathway. *Int. J. Mol. Sci.* 2024, *25*, 6468

**DOI:** 10.3390/ijms27114907

**Published:** 2026-05-29

**Authors:** Yuying Li, Wei Yan, Yu Qin, Liwei Zhang, Sheng Xiao

**Affiliations:** 1 Key Laboratory of Chemical Biology and Molecular Engineering of Education Ministry, Shanxi Key Laboratory of Biotechnology, Institute of Biotechnology, Shanxi University, Taiyuan 030006, China; yanwei978615472@163.com (W.Y.); ty-qiny@kingmed.com.cn (Y.Q.); 2 Key Laboratory of Chemical Biology and Molecular Engineering of Education Ministry, Institute of Molecular Science, Shanxi University, Taiyuan 030006, China; lwzhang@sxu.edu.cn; 3 Department of Pathology, Brigham and Women’s Hospital, Harvard Medical School, Boston, MA 02115, USA; sxiao@rics.bwh.harvard.edu

In the original publication [1], there was a mistake in Figure 4E as published. The second and third images in Figure 4E are overlap, and the third image has been corrected. The corrected Figure 4E appears below. There was a mistake in Figure 5A,B as published. The fourth lane of the p-AKT band is duplicated in Figure 5A, and the correct p-AKT band has been corrected. The quantitative analysis of p-AKT in Figure 5B has been corrected. The corrected Figure 5A,B appears below. There was a mistake in Figure 6A as published. There were image and data annotation errors C2 + L in Figure 6A. The corrected Figure 6A appears below. The authors state that the scientific conclusions are unaffected. This correction was approved by the Academic Editor. The original publication has also been updated.

**Figure 4:**
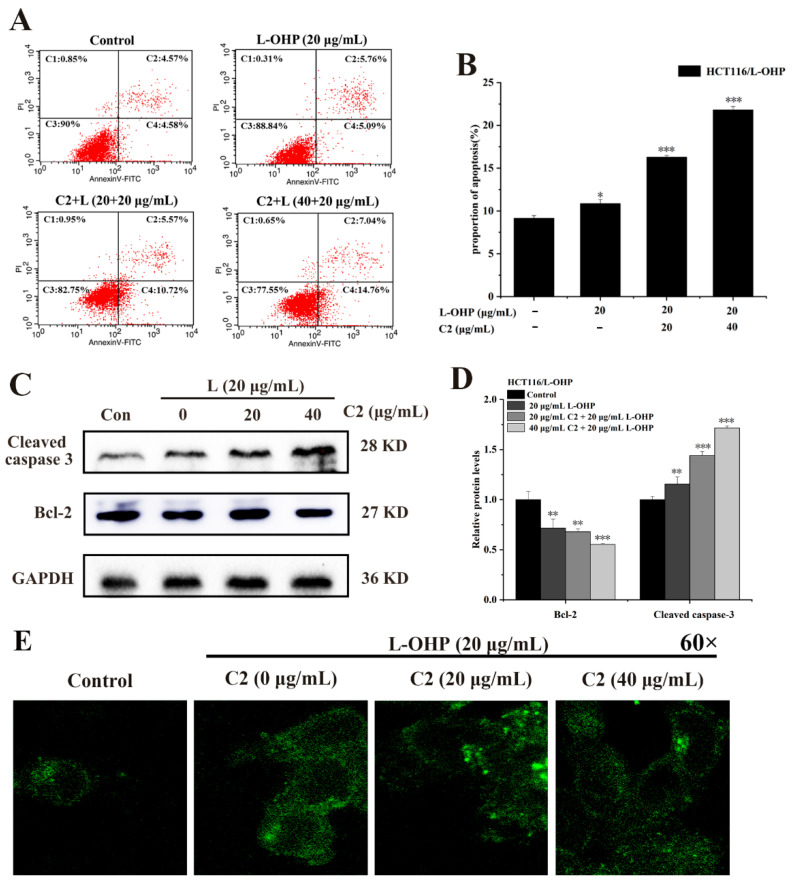
C2 enhanced L-OHP-induced apoptosis and autophagy. (**A**,**B**) Flow cytometry was used to analyze the effect of C2 on L-OHP-induced apoptosis. (**C**,**D**) Expression of Bcl-2 and cleaved caspase 3 was examined by Western blotting analysis. (**E**) MDC staining was performed to analyzed the C2-enhanced L-OHP-induced autophagy by the laser confocal microscope (60×). All data represent the mean ± standard deviation of three independent experiments. * *p* < 0.05, ** *p* < 0.01, *** *p* < 0.001.

**Figure 5:**
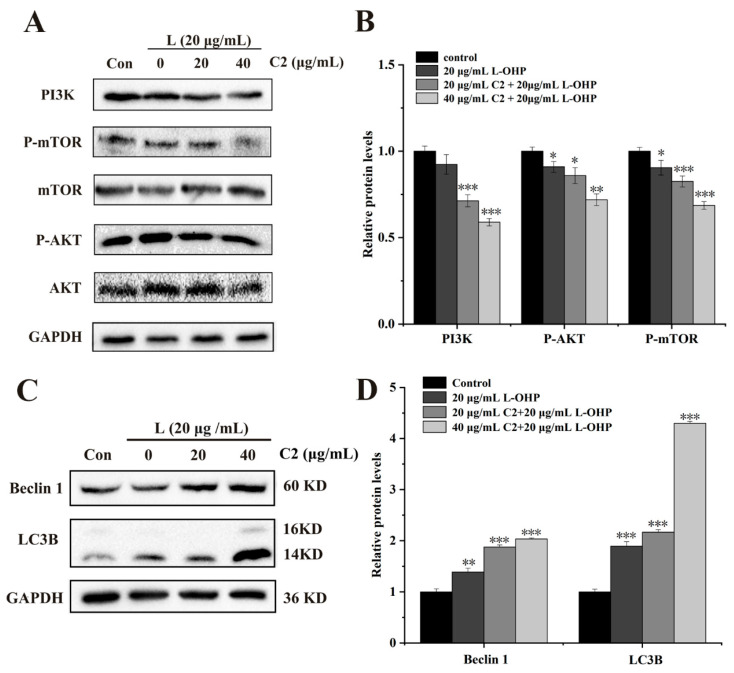
C2 enhances L-OHP-induced autophagy by modulating the PI3K/AKT/mTOR pathway. (**A**,**B**) Expression of PI3K, AKT, mTOR, p-AKT, and p-mTOR was examined by Western blotting analysis. (**C**,**D**) Expression of Beclin 1 and LC3B was examined by Western blotting analysis. All data represent the mean ± standard deviation of three independent experiments. * *p* < 0.05, ** *p* < 0.01, *** *p* < 0.001.

**Figure 6:**
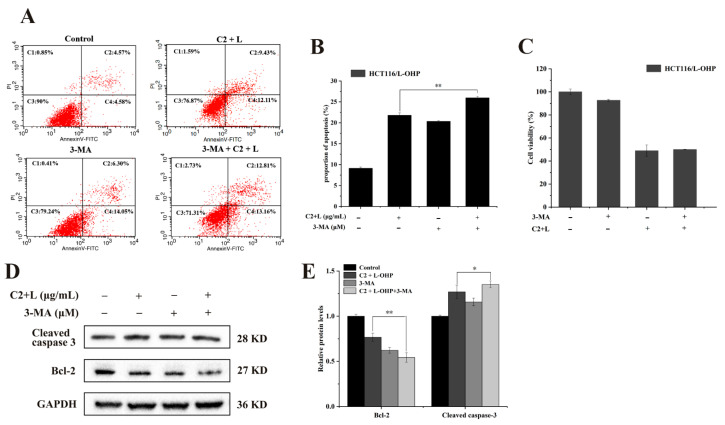
The combination of C2 and L-OHP induced protective autophagy. (**A**,**B**) Flow cytometry was used to analyze the effect of 3-MA (100 μΜ) on C2 + L-OHP-induced apoptosis. (**C**) Cell viability of 3-MA combined with C2 + L-OHP treatment by MTT. (**D**,**E**) Expression of Bcl-2 and cleaved caspase3 was examined by Western blotting analysis after 3-MA combined with C2 + L-OHP treatment. All data represent the mean ± standard deviation of three independent experiments. * *p* < 0.05, ** *p* < 0.01.

## References

[1] Li Y., Yan W., Qin Y., Zhang L., Xiao S. (2024). The Anthraquinone Derivative C2 Enhances Oxaliplatin-Induced Cell Death and Triggers Autophagy via the PI3K/AKT/mTOR Pathway. Int. J. Mol. Sci..

